# Subclinical Hypothyroidism in Children: Natural History and When to Treat

**DOI:** 10.4274/Jcrpe.851

**Published:** 2013-03-01

**Authors:** Gianni Bona, Flavia Prodam, Alice Monzani

**Affiliations:** 1 Università del Piemonte Orientale “A. Avogadro”, Department of Health Sciences, Novara, Italy

**Keywords:** Subclinical hypothyroidism, children, natural history, treatment

## Abstract

Subclinical hypothyroidism (SH) is a quite common disorder in the pediatric age group. The aim of this paper is to present a review of the studies investigating the natural course of SH and the effects of replacement therapy with levothyroxine in childhood. We systematically searched PubMed, Cochrane, and EMBASE (1990 to 2012) and identified 14 articles suitable to be included. SH is a benign process that does not influence anthropometric parameters or puberty onset, and in most cases, it is a remitting disease, with a low risk of development of overt hypothyroidism, more frequently evolving toward euthyroidism or steadily remaining in a condition of isolated hyperthyrotropinemia. Studies analyzing the effects of replacement therapy in SH have reported an increased growth velocity in children with short stature or chronic diseases, discordant effects on thyroid volume reduction, and no effects on neurocognitive function. SH in children and adolescent is often a self-remitting process and its treatment should be considered only when thyroid stimulating hormone values are higher than 10 mIU/L, when clinical signs or symptoms of impaired thyroid function or goiter are detected, or when SH is associated with other chronic diseases.

**Conflict of interest:**None declared.

## INTRODUCTION

**Natural History of Subclinical Hypothyroidism in Children and Adolescents**

Subclinical hypothyroidism (SH) is defined biochemically as a serum thyroid stimulating hormone (TSH) concentration above the statistically defined upper limit of the reference range and a serum free thyroxine (fT4) concentration within its reference range (1). The clinical presentation varies widely, ranging from no manifestations to clear signs or symptoms of hypothyroidism. SH prevalence in the adult population is reported to be 1-10%, being higher in the elderly, in females, and in whites (2,3,4,5). In the pediatric population, SH prevalence is reported to be slightly lower than 2%, although epidemiological studies concerning childhood and adolescence are scanty. Therefore, SH is a quite common disorder in pediatric patients, and both primary care physicians and pediatric endocrinologists frequently face the decision of what to do about these children. 

In order to adequately manage a case of SH, it is of paramount importance to know how this condition will probably evolve and when it should be treated. The natural course of SH in adults seems to progress to overt hypothyroidism (reduced circulating thyroid hormones) in proportions ranging from <1% up to 20% according to different studies (1,3,4), with higher rates of progression in patients with higher baseline serum TSH concentration, elevated anti-thyroid auto-antibodies and higher degree of hypoechogenicity at thyroid ultrasound (6,7). 

We systematically searched PubMed, Cochrane, and EMBASE (1990 to 2012) and identified 8 articles analyzing the natural history of SH in the pediatric age group (8,9,10,11,12,13,14,15)-6 longitudinal trials (8,9,11,12,13,15) and 2 retrospective ones (10,14). Overall, data from a total of 3872 children were reported, even if most studies included a small number of subjects and only one retrospective study included a larger study population (14). Children’s age showed a wide range of variation from 6 months to 19 years. The follow-up duration after the diagnosis of SH varied from a period of 2 years to 9.5 years.

Gopalakrishnan et al (8) found in their study that out of 32 Indian children and adolescents with goitrous autoimmune thyroiditis followed up for a minimum period of 2 years, 7 (21.9%) had their TSH normalized, 21 (65.6%) remained in a condition of SH, and only 4 (12.5%) developed overt hypothyroidism. 

Zois et al (9) reported persistence of SH in all 7 Greek adolescents with autoimmune thyroiditis followed for 5 years. They also showed that thyroid hypoechogenicity, thyroperoxidase antibodies (TPO-Abs) and thyroglobulin antibodies (TG-Abs) increased over time. 

Radetti et al (10) reported in their retrospective multicenter study that during the follow-up, out of 55 Italian children with Hashimoto’s thyroiditis, 16 (29.1%) became euthyroid, while SH persisted in the remaining 39 children - 16 (29.1%) had a TSH value constantly elevated one- to twofold above the upper normal limit and 23 (41.8%) had a TSH value increased to more than twofold above the upper normal limit. Since levo T4 (LT4) treatment was started and patients were excluded from the study as soon as TSH became twofold above the upper normal limit, it was not possible to state how many children actually would have developed an overt hypothyroidism. The authors also reported that the presence of goiter and elevated TG-Abs at presentation and the progressive increase in TPO-Abs and TSH value may be predictive of development of hypothyroidism for the group as a whole, even if none of these parameters may predict the deterioration of thyroid function in individual subjects.

Wasniewska et al (11) followed up for 2 years 92 Italian children with “idiopathic” SH, defined as an incidental finding of elevated TSH, for which all etiological causes of SH had been excluded. They reported that 38 (41.3%) patients normalized their TSH, while 54 (58.7%) remained in SH state - 43 (46.7%) had TSH levels between 5-10 mIU/L and 11 (12%) had increased TSH levels (>10 mIU/L - 10.5-15 mIU/L); none developed overt hypothyroidism. Neither TSH and fT4 values at entry nor the presence of family history of thyroid diseases were predictive of normalization of TSH or persistence of SH. 

Moore (12) studied 18 American patients with autoimmune thyroiditis and elevated TSH concentration, eleven of whom had never been treated; 7 of these patients were followed up after at least 1 year of treatment discontinuation. During the observation period, TSH normalized in 7 subjects, TSH remained elevated and fT4 levels were normal in 10, and only in 1 patient, the increased TSH level was associated with reduced fT4 values. 

In the study by Jaruratanasirikul et al (13), after six years of follow-up, out of 8 Thai girls with SH due to Hashimoto’s thyroiditis, 4 (50%) achieved normal thyroid function without any medication and the other 4 patients developed overt hypothyroidism which was treated with LT4. The authors reported that goiter size remained unchanged without medication, and no clinical or biochemical marker at baseline was able to predict who will become euthyroid or hypothyroid.

Lazar et al (14) in their retrospective multicenter study analyzed a database of 121 052 children aged 0.5-16 years who had a TSH determination in 2002 and who were followed up until 2007. They reported that about 3%, namely 3632 subjects, had SH according to the first TSH determination. During the 5-year follow-up, TSH values tended to normalize in different proportions according to the different degree of initial TSH elevation. In subjects with TSH >5.5 to ≤10 mIU/L, 73.6% normalized their TSH, about 25% maintained their TSH between 5.5 and 10 mIU/L, about 2% had their TSH increased to over 10 mIU/L but with normal fT4, and 0.03% of the subjects developed overt hypothyroidism requiring medical treatment. In subjects with TSH >10 mIU/L, 40% normalized their TSH, 33.1% reduced their TSH to a value between 5.5 and 10 mIU/L, 24.9% maintained their TSH >10 mIU/L, and 0.2% developed overt hypothyroidism requiring medical treatment. Predictive factors for a sustained highly elevated TSH (>10 mIU/L) were an initial TSH greater than 7.5 mIU/L and female gender, whereas age was not found to be a significant predictive factor.

On the whole, from the above-mentioned studies, it may be stated that SH in children and adolescents is a benign and remitting process with low risk of evolution toward overt hypothyroidism, despite the limitation of the low number of patients included in the studies and the high heterogeneity of the study populations. Indeed, most of the subjects included in the studies reverted to euthyroidism or remained in SH state, sometimes with an increase in TSH values. The rate of development of overt hypothyroidism ranged between 0% and 12.5% in three studies (9,10,11), with only one study (13) reporting an evolution toward overt hypothyroidism in half of the 8 investigated children. 

Moreover, in all studies, normal height, body mass index (BMI), and age of puberty were found and no clinical manifestations of hypothyroidism were reported, when analyzed, regardless of the evolution of thyroid function, suggesting that SH natural course does not imply signs of thyroid impairment. 

In the prediction of the natural course of SH, Radetti et al (10) reported that the initial presence of goiter and elevated TG-Abs and the progressive increase in TPO-Abs and TSH value may be predictive of progression toward overt hypothyroidism for the group as a whole, and Lazar et al (14) reported that an initial TSH higher than 7.5 mIU/L and female gender are predictive factors for a sustained highly elevated TSH.

Finally, a special mention should be given to the results reported by Leonardi et al (15) who observed a high persistence of SH in children with “false positive” results at neonatal screening for congenital hypothyroidism. They studied a group of Italian children “false positive” at neonatal screening for congenital hypothyroidism, who had normal fT4 and normal or slightly elevated TSH values at recall examination at a mean age of 22 days. When re-tested at 2-3 years of age, 28 of them had SH (TSH 4-10.1 mIU/L with normal fT4). Twenty of these 28 children were treated with replacement therapy and then withdrawn from therapy 2-3 months before re-evaluation. Out of the 28 children with SH, at 4.1-6.6 years of age, TSH was normal in 9 children (32%) and persisted elevated in the remaining 19 (68%), ranging between 4.0 and 9.2 mUI/L. At 7.2-9.5 years of age, TSH was still normal in the 9 children who previously normalized their thyroid function, became normal in 5/19 of the children with the previous elevated TSH value, and persisted above normal in the remaining 14/19, ranging between 4.1-8.2 mUI/L. On the whole, half of the children (14/28) reverted to euthyroidism at the end of the observation period, half remained in SH state, and none developed overt hypothyroidism. Studying thyroid morphology, Leonardi et al showed the presence of hemiagenesis, hypoplasia of one lobe or goiter in half of these children and detected TPO mutation in one child and TSH receptor (TSH-R) mutation in another child. Therefore, the authors concluded that a mild hyperthyrotropinemia at neonatal screening, in particular when this data is confirmed in further evaluations, may be suggestive of congenital anatomic or functional anomalies of the thyroid gland, even if their clinical significance is not clear since none of the studied children developed overt hypothyroidism during the follow-up.

**Effects of Replacement Therapy for Subclinical Hypothyroidism in Children and** **Adolescents**

The decision to treat SH or not should take into account both the risk of evolution toward overt hypothyroidism and the possible consequences of hyperthyrotropinemia itself, because in adults, an increased risk of cardiovascular diseases (16,17), depression (18), dyslipidemia (19), increased serum prolactin concentrations (20), and negative influence on the hemostatic profile (21) have been reported. On the other hand, the adverse effects of overtreatment, such as reduced bone mass (22,23) and heart rhythm disorders (24), should be likewise considered. 

We systematically searched PubMed, Cochrane, and EMBASE (1990 to 2012) and identified 6 articles investigating the possible effects of LT4 replacement therapy in SH (25,26,27,28,29,30) (Table 1). Three were retrospective trials (26,28,29), one of them being a case-control study (26), and 3 were longitudinal trials (25,27,30), two of them being cross-over studies [in one of them, LT4 was compared with placebo (27) and in the other one - with no treatment (30)]. Overall, data from a total of 141 children were reported. Children’s age varied between 3.6 and 18.5 years. The treatment duration varied widely, ranging from 6 weeks to 12 years. The LT4 dosage varied between 2 and 4 μg/kg/day, was not available in two studies, and in one study, the dosage per kilogram body weight was not reported.

In 3 studies, the effects of replacement treatment on growth were analyzed (25,26,27). The longitudinal study by Cetinkaya et al (25) included 24 pre-pubertal and 15 pubertal Turkish children complaining of short stature, in which SH was diagnosed by TSH-releasing hormone stimulation test. They received LT4, 2 μg/kg/day, for 1 year. After 6 months and 1 year of treatment, growth velocity and growth velocity standard deviation score (SDS) were found to be significantly increased in both pre-pubertal and pubertal subjects, even if the improvement was more significant in the pubertal group. The authors reported no signs of clinical hyperthyroidism in any patient. 

In a retrospective study by Chase et al (26), 25 American children with SH and type 1 diabetes were matched to 25 diabetic controls. Five of the 25 cases were pubertal and 20 pre-pubertal. The 25 diabetic children with SH were given LT4 in doses of 2-4 mg/kg/day for 2 years, with TSH and T4 determinations at least twice yearly to adjust the dosage in order to keep their levels in normal range. The authors reported a significant improvement in growth velocity after LT4 treatment in pre-pubertal children compared with diabetic controls, the improvement being more significant in children with more elevated TSH values. No significant difference was found in growth velocity between pubertal children and diabetic controls and in height Z-scores between all treated children and diabetic controls.

In the longitudinal study by Eyal et al (27), 8 American patients with borderline thyroid function and Fanconi anemia were randomly assigned to first receive LT4 for 7 months followed by 7 months of placebo, or to start with placebo followed by LT4. The starting dose was 3 mg/kg/day. A faster growth velocity was observed in all subjects during treatment compared with placebo. However, clinical symptoms of overtreatment were reported in 12.5% of treated children. 

On the whole, all these three studies reported an increased growth velocity in treated children, even if it has to be taken into account that SH was not an isolated condition but was diagnosed in the context of short stature in one case (25) and was a co-morbidity with other chronic diseases in the other two cases (26,27).

In 2 studies, the effects of replacement treatment on thyroid volume were analyzed (28,29). Svensson et al (28) in their retrospective study analyzed data on 42 Swedish children with SH and autoimmune thyroiditis who were given LT4. A significant reduction in median thyroid volume SDS was seen at each 2-year assessment during therapy. Significant correlations were found between reduction in thyroid volume SDS and thyroid volume SDS at baseline, treatment duration, and TSH/T4 levels at baseline. No complications with LT4 treatment were reported during the study period.

The retrospective study by Rother et al (29) reported the effects of LT4 in 16 American children with autoimmune thyroiditis treated for a period ranging from 1 to 12 years. In 75% of them, no change in thyroid volume was observed, whereas in the remaining 25%, a reduction in goiter size took place.

The contrasting results by these two studies may firstly depend on the different age at diagnosis of the study populations. In fact, the mean age at diagnosis of the children in the study by Rother et al (29) was higher than that of the children in the study by Svensson et al (28). In particular, the subjects in the former study were peri-pubertal, and it is well known that a rapid increase in thyroid volume normally takes place during puberty (31). Therefore, the lack of reduction in thyroid size could be due to the more intense thyroid growth in peri-pubertal subjects. Moreover, it should be noted that the estimation of thyroid volume was made by palpation in one study (29) and by ultrasonography in the other one (28). 

Finally, only one study investigated the effect of LT4 on neurocognitive function (30). Ajiaz et al (30) reported no effects on neuropsychological functions during the 6-8 week observation period. In particular, no improvement in attention problems was found, however, it has to be stated that the attention problems observed in SH children as compared to healthy controls were only detected by neuropsychological tests and never previously reported by parents. One possible explanation for the lack of effects found is the low number of children analyzed and the short intervention period. It is possible that a longer period of being on or off LT4 therapy may have produced different results.

The rate of development of overt hypothyroidism ranged between 0% in three studies (9-11) to 12.5%, with only one study (13) reporting an evolution toward overt hypothyroidism in half of the 8 investigated children.

In conclusion, the decision about treatment of SH in children and adolescents is still a matter of debate. None of the consensus statements published about the management of SH (1,32) addressed the issue of SH in the pediatric population. However, according to the available evidence in children, which is limited, SH seems to be a self-limiting condition with a low rate of progression to overt hypothyroidism. Therefore, treatment of SH in children should be considered only when TSH values are higher than 10 mIU/L, when clinical signs or symptoms of impaired thyroid function or goiter are detected, or when SH is associated with other chronic diseases. In children with SH but with no goiter, negative anti-thyroid antibodies and a TSH level of 5-10 mIU/L, replacement therapy is not justified, both because of the low risk to develop overt hypothyroidism and because they could simply be euthyroid outliers, representing 2.5% of normal individuals whose TSH values are above the 97.5th percentile of euthyroid distribution. Finally, when the decision to start LT4 therapy is taken, clinicians should know that hyperthyroidism due to overtreatment of SH has been reported to be an infrequent condition in pediatric patients.

## Figures and Tables

**Table 1:**
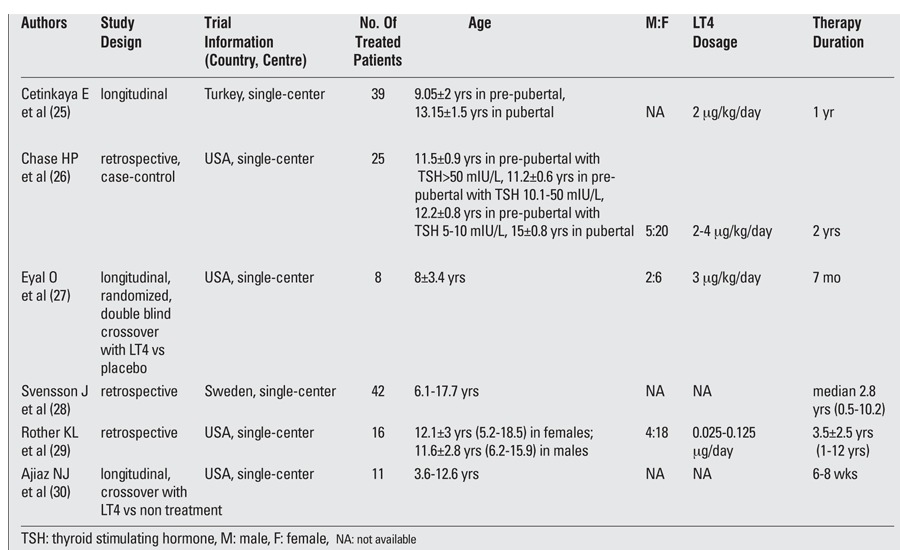
Studies evaluating the effects of levo thyroxine (LT4) replacement therapy in children with subclinical hypothyroidism
